# The SCMCIE94 Protocol for Countries With Limited Resources

**DOI:** 10.1200/JGO.19.00044

**Published:** 2019-04-18

**Authors:** Ian J. Cohen

**Affiliations:** ^1^ Schneider Children’s Medical Center of Israel, Elkanah, Israel; ^2^ Tel Aviv University, Tel Aviv, Israel

## TO THE EDITOR:

Majeed et al^1^ are to be admired for their intellectual honesty in presenting their experience with Ewing sarcoma in their country. This first essential step in improving treatment results is a solid basis for better subsequent results. The next step is to use a unified affordable protocol and stick to it to enable the results to be analyzed in such a way that they can be used to build on in the future. Because we were dismayed by our previous results for Ewing sarcoma, we looked for ways of improving treatment without increasing cost. By adopting the elements of therapy that had been useful in different published protocols, we were able to have an impact on the outcome, especially in isolated limb Ewing sarcoma. The protocol SCMCIE94 (Schneider Children's Medical Center of Israel Ewing 1994 protocol) we developed did not include any expensive new drugs.^2^ We did not give granulocyte colony-stimulating factor after we found that although it increased the white cell count, it did not shorten recovery time after chemotherapy because of a lack of effect on platelets. We were able to improve long-term results especially by preventing early relapse in these patients, and even though we could not pinpoint the reason for our better results, we suspect that the radiotherapy before and after surgery may well have been the critical intervention. Although the protocol did not affect outcome in metastatic Ewing sarcoma, it did prolong the relapse-free period, which may have significance for future protocols.
